# ABO Incompatibility between the Mother and Fetus Does Not Protect against Anti-Human Platelet Antigen-1a Immunization by Pregnancy

**DOI:** 10.3390/jcm11226811

**Published:** 2022-11-17

**Authors:** Laila Miserre, Sandra Wienzek-Lischka, Andreas Mann, Nina Cooper, Sentot Santoso, Harald Ehrhardt, Ulrich J. Sachs, Gregor Bein

**Affiliations:** 1 Institute for Clinical Immunology, Transfusion Medicine and Hemostasis, Justus-Liebig-University, Langhansstr. 7, 35392 Giessen, Germany; 2 German Centre for Feto-Maternal Incompatibility, University Hospital Giessen and Marburg, Campus Giessen, Langhansstr. 7, 35392 Giessen, Germany; 3 Department of General Pediatrics and Neonatology, Justus-Liebig-University, Feulgenstr. 10-12, 35392 Giessen, Germany; 4 Department of Thrombosis and Haemostasis, University Hospital Giessen and Marburg, Campus Giessen, Langhansstr. 2, 35392 Giessen, Germany

**Keywords:** fetal and neonatal alloimmune thrombocytopenia, ABO blood group, anti-human platelet antigen-1a

## Abstract

(1) Background: ABO blood group incompatibility between the mother and fetus protects against anti-D immunization by pregnancy. The possible role of ABO incompatibility in protecting against anti-human platelet antigen-1a immunization is unclear. (2) Methods: This study retrospectively screened 817 families (mother-father-neonate trios) of suspected fetal and neonatal alloimmune thrombocytopenia for inclusion. ABO genotypes were determined in 118 mother-child pairs with confirmed alloimmune thrombocytopenia due to anti-HPA-1a antibodies, and 522 mother-child pairs served as the control group. The expression of blood group antigen A on platelets was determined in 199 consecutive newborns by flow cytometry and compared with adult controls. (3) Results: ABO incompatibility between mother and fetus did not protect against anti-human platelet antigen-1a immunization by pregnancy. ABO blood groups of mothers and/or fetuses were not associated with the severity of fetal and neonatal alloimmune thrombocytopenia. The expression pattern of blood group A antigens on the platelets of newborns mirrored that of adults, albeit on a lower level. Blood group A antigen was detected on a subpopulation of neonatal platelets, and some newborns revealed high platelet expression of A determinants on all platelets (type II high-expressers). (4) Conclusion: The lack of a protective effect of ABO incompatibility between mother and fetus against anti-human platelet antigen-1a immunization by pregnancy may indicate that fetal platelets are not the cellular source by which the mother is immunized.

## 1. Introduction

Fetal and neonatal alloimmune thrombocytopenia (FNAIT) is caused by maternal antibodies against fetal platelet antigens inherited from the father. Placental transport of immunoglobulin G class antibodies from the maternal to the fetal circulation may lead to opsonization of fetal platelets resulting in thrombocytopenia and bleeding complications (for review see [1]). In Caucasian populations, most cases of severe FNAIT are caused by antibodies directed at human platelet antigen (HPA)-1a [2]. The incidence of FNAIT is 1 in 1000 pregnancies and the incidence of its most severe complication, intracranial hemorrhage (ICH) leading to intrauterine death or long-term neurologic sequelae, occurs in 1 of 10,000 pregnancies [3].

The etiology of FNAIT equals those of hemolytic disease of the fetus and newborn (HDFN), where maternal antibodies against fetal blood group antigens may lead to fetal red blood cell opsonization resulting in fetal anemia, in severe cases, hydrops, and fetal demise. HDFN is most frequently caused by maternal anti-D antibodies. An RhD-negative mother is immunized by fetal transplacental hemorrhage of D-positive red blood cells during pregnancy and a larger volume at delivery (for review see [4]). Levine was the first to observe that in matings of RhD-negative mothers with HDFN, the incidence of incompatible ABO blood group matings was lower than expected [5]. The protective action of ABO incompatibility on anti-D immunization was confirmed in subsequent studies [6]. Recently, Zwiers et al. corroborated that ABO incompatibility protects against non-D red blood cell alloimmunization by pregnancy [7].

A small study of 25 FNAIT cases suggested that ABO incompatibility between the mother and fetus protects similarly against HPA-1a immunization by pregnancy [8]. In all 25 FNAIT cases, the mother and child were ABO compatible. The authors of this study concluded that fetal platelets must express ABO antigens, and fetal platelets may be cleared from the maternal circulation in cases of ABO-incompatible pregnancies before immunization of the mother occurs. Two studies investigated the possible association of the maternal ABO blood group with FNAIT severity [9,10]. Both studies reported a similar maternal ABO blood group distribution in FNAIT cases and controls. However, the possible protective role of ABO incompatibility between the mother and fetus on the incidence of anti-HPA-1a immunization by pregnancy was not investigated in either study. Ahlen et al. observed an association of severe FNAIT (neonatal platelet count <50 × 10^9^/L) with maternal blood group A [10].

Given these conflicting findings, we investigated (1) whether ABO incompatibility between the mother and fetus protects against anti-HPA-1a immunization by pregnancy and (2) whether ABO blood groups of the mother and/or the fetus are associated with FNAIT severity in a cohort of 817 families of suspected FNAIT. Furthermore, we investigated the expression of the blood group A antigen on platelets of 199 consecutive newborns. This is the first systematic study on blood group A antigen expression on platelets in neonates.

## 2. Materials and Methods

### 2.1. Patients and Case Definitions

**Figure 1:**
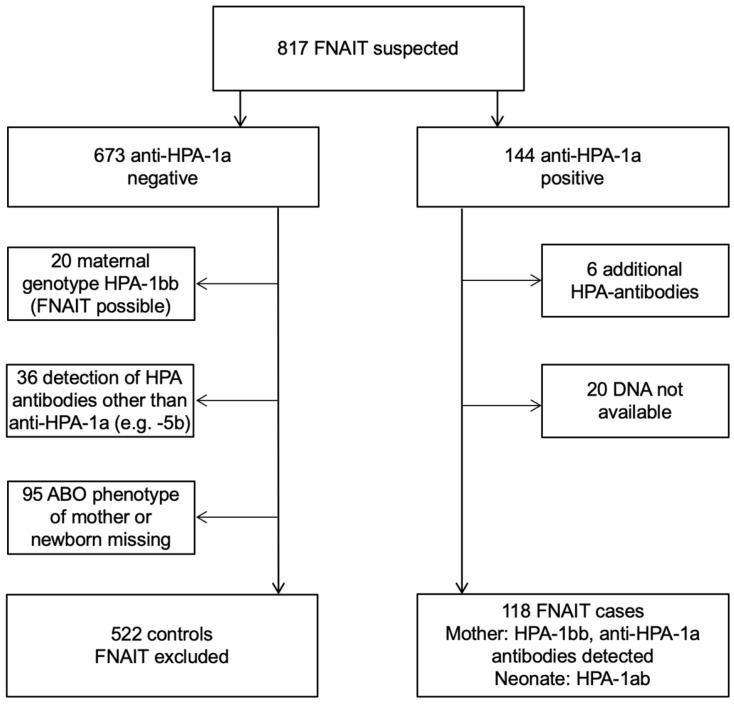
Screening of 817 families of suspected FNAIT. Definition of controls and cases.

This study retrospectively screened 817 families (mother-father-neonate trios) of suspected FNAIT, referred to our Centre for Feto-maternal Incompatibility between January 2000 and April 2016 for inclusion (Figure 1). Confirmed FNAIT cases were HPA-1bb mothers who had serological detection of anti-HPA-1a antibodies and were delivered by an HPA-1ab neonate. FNAIT cases with antibodies other than anti-HPA-1a (e.g., anti-HPA-5b) were excluded from the study group because of differences in clinical FNAIT presentation, such as higher neonatal platelet counts in FNAIT cases due to anti-HPA-5b compared with FNAIT cases due to anti-HPA-1a [2]. Mothers with additional anti-HPA-antibodies (mainly anti-HPA-5b) besides anti-HPA-1a were also excluded. Controls included mothers without detection of anti-HPA antibodies. Furthermore, possible FNAIT cases of HPA-1bb mothers without detectable HPA-1a antibodies at the time of post-partum blood sampling were excluded from controls. Finally, restricted by lack of material, we included 118 mother-child pairs with FNAIT due to anti-HPA-1a antibodies. Additionally, 522 mother-child pairs of suspected FNAIT where FNAIT was excluded served as the control group. Clinical data were retrieved from the in-house laboratory information system and medical records, including the referring physician’s letter.

### 2.2. Work-Up of FNAIT Families

Platelet counts were determined in ethylenediaminetetraacetic acid (EDTA)-anticoagulated whole blood using a hematology analyzer (KX-21N, Sysmex Corporation, Kobe, Japan). Platelet counts <10 × 10^9^/L were controlled microscopically in a counting chamber. ABO blood groups of all suspected cases were determined following routine standards for adult and neonatal pretransfusion testing. The diagnosis of HPA genotypes and detection of anti-HPA antibodies are described elsewhere [11].

### 2.3. ABO Genotyping in FNAIT Cases (Mother and Newborn)

ABO*A1 and ABO*A2 alleles and hetero- and homozygosity of major *ABO* alleles were discriminated by performing genotyping with in-house TaqMan real-time PCR assays (TaqMan; applied biosystems/ThermoFisher Scientific, Waltham, MA, USA), to detect the major *ABO* alleles ABO*A1.01, ABO*A2.01, ABO*B.01, ABO*O.01, and ABO*O.02 according to the International Society of Blood Transfusion blood group allele database (*ABO* blood group alleles v1.1 171023; Supplementary Table S1). Inconclusive genotyping results were resolved using PCR with sequence-specific primers (PCR-SSP; inno-train, Kronberg, Germany).

### 2.4. Flow Cytometric Measurement of A Antigens on Adult and Neonatal Platelets

The following procedure was used for flow cytometric measurement of A antigens on adult and neonatal platelets. First, 5 mL of EDTA anticoagulated cord blood samples (newborns) or blood samples (adults) was added to 6 mL 0.5 g% EDTA/NaCl buffer and centrifuged for 16 min. at 250× *g* (w/o brake). Next, 3 mL of platelet-rich plasma was harvested and mixed with 4 mL NaCl (pH 6.5), followed by 2 washing steps (1200× *g*, 10 min). The final pellet was carefully resuspended in 1 mL phosphate-buffered-saline (PBS)-EDTA (supplemented with prostaglandin E1), and the concentration of platelets was measured (hematology analyzer KX-21N, Sysmex Corporation, Kobe, Japan) and adjusted to 5 × 10^7^/mL. Then, 10 µL of this suspension was added to 30 µL PBS and stained with anti-CD41a (final concentration 0.25 µg/mL) and anti-A or isotype control antibody, respectively (final concentration 1 µg/mL), in a final volume of 50 µL for 30 min. at room temperature. Without further centrifugation, staining was adjourned by adding 700 µL PBS, and flow cytometric analysis was conducted within 2 h. Stained platelets were analyzed by flow cytometry (FACS Canto II, BD Biosciences, Franklin Lakes, NJ, USA; FACSDiva software version 8.01). Finally, 30,000 events were counted, and platelets were gated by forward/side scatter and staining for CD41a. The distribution of the anti-A signal (median fluorescence intensity) on CD41a positive cells was statistically evaluated. A cut-off for positive anti-A staining was defined by a window that included ≤1% positive events within the specimen stained in parallel with irrelevant isotype control antibody. We did not investigate the expression of B antigen since the phenotype of blood group B is only 12% in this population (Table 1). Furthermore, the expression pattern of A and B antigens on adult platelets is similar [12]. Newborns with known HDFN were excluded. All adult and newborn cohort samples were analyzed prospectively on alternating days within 4 months.

### 2.5. Antibodies

The antibodies used in this study were mouse anti-A: IgG1, kappa, phycoerythrin-conjugated; Clone: BRIC 145; International Blood Group Reference Laboratory, Bristol, UK. Mouse isotype control: IgG1, kappa, phycoerythrin-conjugated; Clone MOPC-21; BioLegend, San Diego, CA, USA. Mouse anti-human CD41a: IgG1, kappa, APC conjugated; Clone HIP8; ThermoFisher Scientific, Waltham, MA, USA.

### 2.6. Statistical Analysis

Data were managed using Excel (Microsoft Office 365; Microsoft Corporation, Redmond, WA, USA) and analyzed using IBM SPSS Statistics Version 25 for Windows (IBM, Armonk, NY, USA). The graphical illustration was performed with the Prism 8 software package (GraphPad Software, Inc., San Diego, CA, USA). Groups’ characteristics are presented as medians and interquartile ranges (IQRs).

ABO phenotype frequencies were compared between FNAIT cases and 45,295 first-time blood donors using a Chi-square test. Proportions of ABO-compatible pregnancies were compared between 118 FNAIT cases and 522 controls. The effect of ABO phenotypes on the occurrence of ICH, magnitude of thrombocytopenia, and birth weight was assessed using a two-sided Fisher’s exact test and Pearson Chi-square test, Kruskal–Wallis test, and Welch-ANOVA, respectively. Effects of hetero-or homozygosity for alleles ABO*A1.01, ABO*O.01/O.02, and fetomaternal ABO compatibility on the occurrence of ICH, the magnitude of thrombocytopenia, and birth weight were evaluated using the two-sided Fisher’s exact test, Mann–Whitney test, and *t*-test, respectively. A *p* value < 0.05 was considered significant. Missing values are depicted in each figure, if applicable.

## 3. Results

### 3.1. ABO Phenotype Frequencies Do Not Differ between FNAIT Cases and Controls

ABO phenotype frequencies of cases (mothers and neonates) were compared with the ABO phenotype frequencies among 45,295 first-time blood donors to test the hypothesis that the maternal propensity for alloimmunization against HPA-1a is associated with blood groups (Table 1). The differences were not statistically significant.

### 3.2. ABO Incompatibility between the Mother and Fetus Does Not Protect against Anti-HPA-1a Immunization by Pregnancy

**Figure 2:**
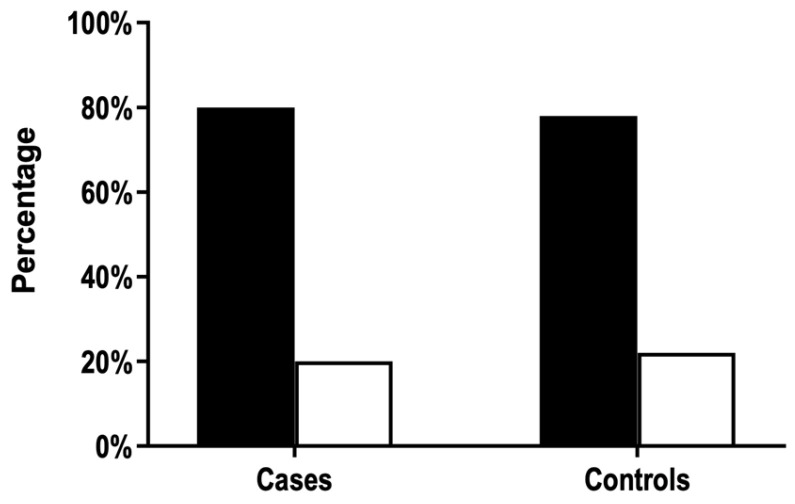
Distribution of ABO-compatible (black bars) and ABO-incompatible (white bars) pregnancies among cases (*n* = 118) and controls (*n* = 522) (Chi-square test, *p* > 0.05).

The proportion of ABO-incompatible pregnancies did not differ between cases and controls (Figure 2). Thus, we did not confirm the hypothesis that ABO incompatibility protects against anti-HPA-1a immunization by pregnancy. In this case, the proportion of incompatible pregnancies would be lower in FNAIT cases.

### 3.3. Maternal ABO Phenotypes Are Not Associated with FNAIT Severity

**Figure 3:**
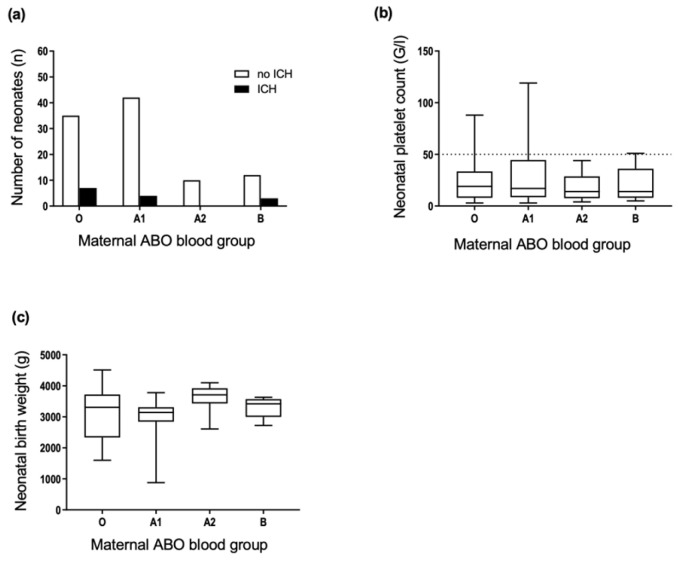
Distribution of maternal ABO phenotypes and the occurrence of (**a**) ICH, (**b**) platelet count nadir, and (**c**) birth weight in neonates of anti-HPA-1a immunized mothers. Dotted line in (**b**) threshold of severe thrombocytopenia, platelet count <50 × 10^9^/L. (**a**) *n* = 113, two-sided Fisher’s exact test, *p* = 0.35; (**b**) *n* = 110, Kruskal–Wallis test, *p* = 0.85; (**c**) *n* = 64, Welch-ANOVA test, *p* = 0.066.

We tested the hypothesis that maternal blood groups may affect the neonatal outcome in cases of FNAIT. The comparison of maternal ABO phenotypes and the occurrence of neonatal ICH revealed no significant associations (Figure 3a). There were no significant associations between the neonatal platelet count (Figure 3b), neonatal birth weight (Figure 3c), and the maternal ABO phenotype. The cases with maternal blood group AB were excluded due to the low number of individuals.

### 3.4. Maternal Gene Dose of the ABO*A1.01 Allele Is Not Associated with FNAIT Severity

**Figure 4:**
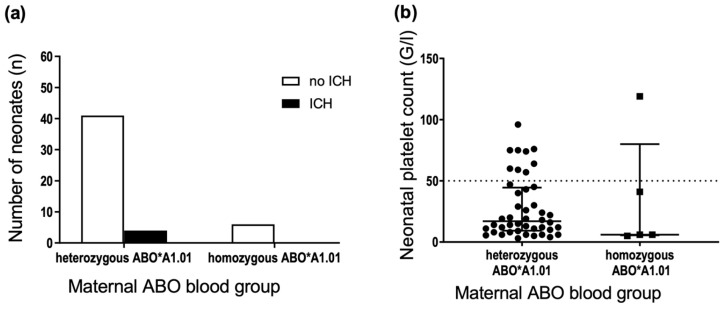
Comparison of maternal ABO*A1.01 hetero- or homozygosity and the occurrence of (**a**) ICH and (**b**) platelet count nadir in neonates of anti-HPA-1a immunised mothers. Dotted line in (**b**) threshold of severe thrombocytopenia, platelet count <50 × 10^9^/L. (**a**) *n* = 51, two-sided Fisher’s exact test, *p* = 1.000; (**b**) Mann–Whitney test, *p* = 0.536, median and interquartile range displayed.

According to a study by Ahlen et al. [10], the gene dose of the ABO*A1.01 allele was associated with FNAIT severity. Among blood group A mothers, the frequency of newborns with severe FNAIT (neonatal platelet count <50 × 10^9^/L) was lower in pregnancies where the mother carried only one ABO*A1.01 allele and higher where mothers carried two ABO*A1.01 alleles. Mothers were stratified according to zygosity for A and O alleles (ABO*A1.01 and ABO*O.01 alleles) to analyze the possible association between maternal ABO genotype and neonatal outcomes. For alleles ABO*A2.01, ABO*O.02 and ABO*B.01, the number of homozygous mothers was too small for valid statistics. There was no significant difference in the incidence of ICH in neonates suffering from FNAIT born to mothers that were hetero- or homozygous for ABO*A1.01 (Figure 4a) or ABO*O.01 (data not shown). Similarly, we observed no significant difference in the platelet count nadir (Figure 4b) in neonates suffering from FNAIT born to mothers that were hetero- or homozygous for ABO*A1.01 or ABO*O.01 (data not shown).

### 3.5. Neonatal ABO Phenotypes Are Not Associated with FNAIT Severity

**Figure 5:**
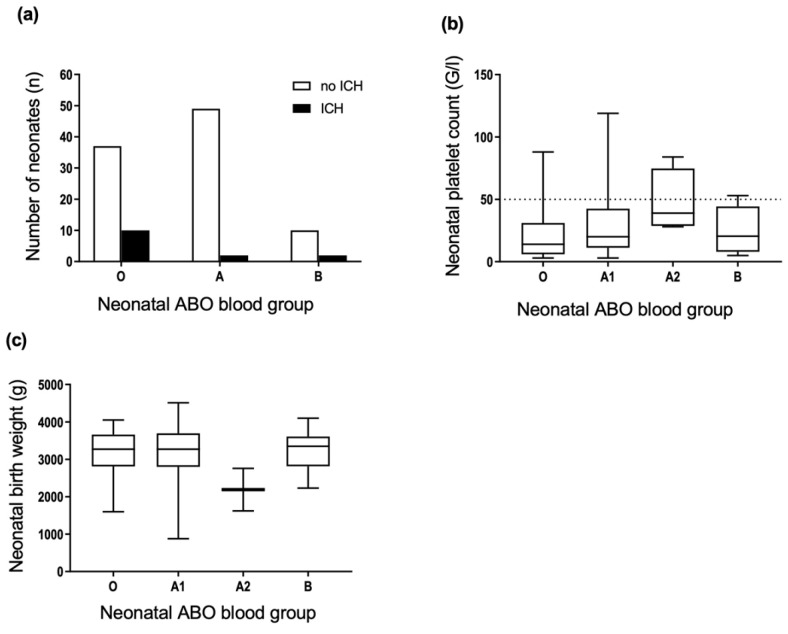
Comparison of neonatal ABO phenotypes and the occurrence of (**a**) ICH, (**b**) platelet count nadir, and (**c**) birth weight. Dotted line in (**b**) threshold of severe thrombocytopenia, platelet count <50 × 10^9^/L. (**a**) *n* = 110, Chi-square test, *p* = 0.035; (**b**) *n* = 107, Kruskal–Wallis test (4) *p* = 0.067, median and interquartile range displayed; (**c**) *n* = 64, Welch-ANOVA, *p* = 0.55, median and interquartile range displayed.

We analyzed the possible association between neonatal ABO phenotype and neonatal outcomes. Results showed a significant difference in the incidence of ICH in newborns stratified according to ABO phenotype. Further tests revealed that ICH occurred significantly more often in neonates with phenotype O than phenotype A (Chi-square test, *p* = 0.035) (Figure 5a). Ten of 47 neonates with blood group O suffered from ICH, compared with 2 of 51 neonates with blood group A. To replicate this association, we evaluated an independent cohort of suspected FNAIT cases with the following inclusion criteria: period 1991–1999; ICH of the fetus or newborn; mother HPA-1bb; maternal anti-HPA-1a antibody detected (no other HPA antibodies); newborn HPA-1ab; ABO blood group determined. 4 of 10 (40%) newborns were blood group O. Thus, the association of blood group O with ICH in the initial cohort could be due to a type 1 error. There were no significant differences in the platelet count nadir and birth weight (Figure 5b,c) between newborns grouped according to ABO phenotype.

### 3.6. ABO Incompatibility between the Mother and Fetus Is Not Associated with FNAIT Severity

**Figure 6:**
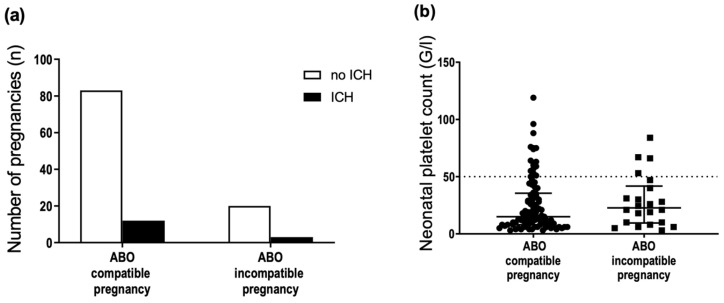
Comparison of fetomaternal ABO compatible and incompatible pregnancies and occurrence of (**a**) ICH, and (**b**) platelet count nadir in neonates. Dotted line in (**b**) threshold of severe thrombocytopenia, platelet count <50 × 10^9^/L. (**a**) *n* = 118, two-sided Fisher’s exact test, *p* = 1.00; (**b**) *n* = 115, Mann–Whitney test, *p* = 0.40, median and interquartile range displayed.

ABO incompatibility between the mother and fetus was not associated with the occurrence of ICH (Figure 6a) and neonatal platelet count (Figure 6b). The possible association of ABO incompatibility with birth weight was not analyzed because of the small sample size. This analysis was repeated with a stricter definition of ABO incompatibility: mother, predicted blood group O, and neonate, predicted blood group A_1_. Employing this strict definition of ABO incompatibility, no association between ABO incompatibility and the occurrence of ICH or neonatal platelet count was found (data not shown).

### 3.7. Blood Group A Antigens Are Weakly Expressed on Newborn Platelets but Strongly Expressed on Platelets of Some Newborns

**Figure 7:**
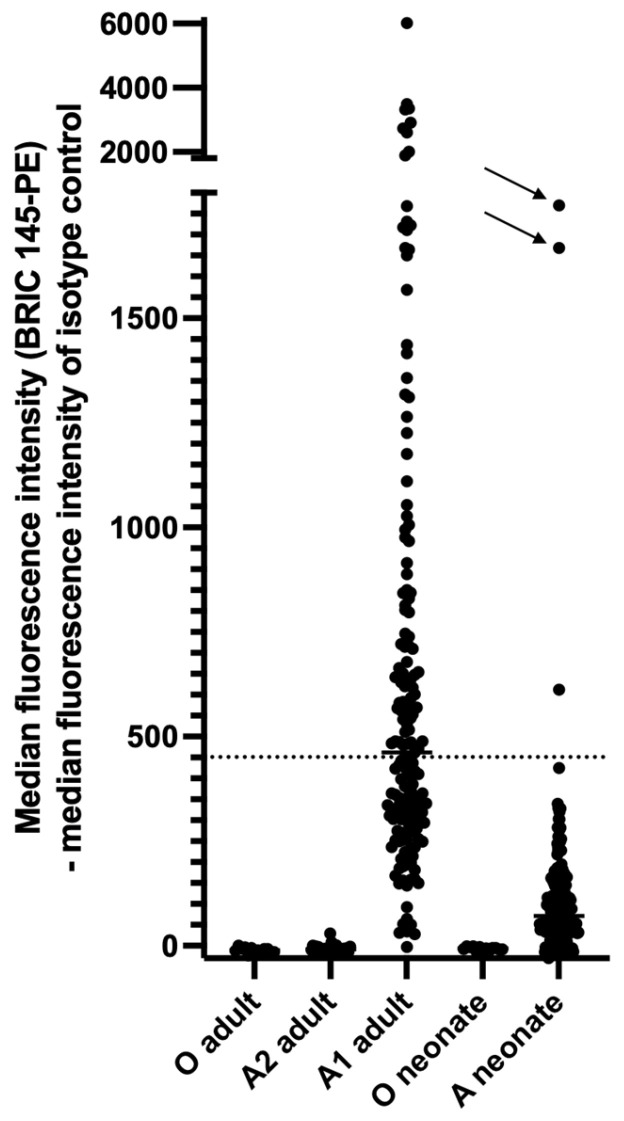
Flow cytometric analysis of blood group A antigen expression by binding of phycoerythrin (PE)-conjugated moAb BRIC 145 on adult blood group O platelets (*n* = 13), adult blood group A_2_ platelets (*n* = 31), adult blood group A_1_ platelets (*n* = 181), neonatal blood group O platelets (*n* = 13), and neonatal blood group A platelets (*n* = 199). *Y*-axis: median fluorescence intensity (MFI) (BRIC 145-PE)–MFI of isotype control. The dotted line represents the mean fluorescence intensity of neonatal blood group A platelets + 2 SD. Arrows: neonatal type II high-expressers.

First, we replicated the findings of Curtis et al. [12] and others regarding A antigen expression on adult platelets (Figure 7). The binding of the monoclonal anti-A antibody BRIC 145 on adult blood group A_2_ platelets could not be distinguished from binding on adult blood group O platelets. An analysis of anti-A-stained platelets of adult blood group A_1_ donors by flow cytometry demonstrated broad histograms that overlapped the histograms of blood group O and blood group A_2_ platelets. According to the definition of Ogasawara et al. [13] 7 of 169 (4.14%) donors were categorized as high-expresser phenotypes (mean of median fluorescence intensities +2 SD). Platelets from one of these 7 donors demonstrated a sharp histogram peak with high A antigen expression on all platelets (type II high-expresser phenotype).

The expression level of blood group A antigens on neonatal platelets was significantly lower compared with adult platelets of blood group A_1_ (Figure 7). The median fluorescence intensity (MFI) of adult blood group A_1_ platelets was 486.0 (95% CI of median 417.0–569.0, *n* = 169) compared with 94.0 (95% CI of median 84.0–112.0, *n* = 199) of neonatal blood group A platelets (*p* < 0.0001, Mann–Whitney test). All but three newborns demonstrated an MFI of anti-A staining below the median MFI of anti-A staining of adult platelets. The histograms of neonatal blood group A platelets broadly overlapped the histograms of neonatal group O platelets. Three newborns were categorized as high-expressers (mean of median fluorescence intensities +2 SD); two demonstrated a sharp histogram peak with high A expression on all platelets (type II high-expresser phenotype). ABO expressor traits influence quantitative ABO(H) expression on platelets, red blood cells and soluble plasma proteins [14]. Due to the blinded design of our study, information about platelet count or signs of hemolysis in the newborns with high-expresser phenotype was not available.

We compared the proportion of adult and neonatal platelets that exhibited anti-A antibody binding above the pre-defined cut-off (see material and methods). Of these, 40.9% (median, 95% CI of median 37.1–44.0; *n* = 169) of adult blood group A_1_ platelets and 15.5% (median, 95% CI of median 13.2–16.5; *n* = 199) of neonatal blood group A platelets were blood group A antigen-positive. The proportion of adult platelets exhibiting anti-A antibody binding did not differ between blood group O and A_2_ platelets.

## 4. Discussion

In a large cohort of well-characterized FNAIT cases, we did not confirm the initial observation by Gratwohl and Shulman [8] that ABO incompatibility between the mother and fetus protects against anti-HPA-1a immunization by pregnancy. Furthermore, ABO blood groups of mothers and/or fetuses were not associated with FNAIT severity. The propensity for the mother to develop anti-HPA-1a antibodies is closely linked to the expression of HLA-DRB3*01:01. Since almost all mothers in this cohort were HLA-DRB3*01:01 positive [15], we did not stratify the groups of this study according to the presence or absence of HLA-DRB3*01:01.

Adult platelets express A and B blood group antigens [16], which are synthesized within the platelet or platelet precursor and are passively absorbed to a minor extent [17,18]. Blood group A and B determinants are expressed on platelet glycoproteins (GP) IIa, IIIa, Ib [19], IIb [20], IV, V, [21], CD109, PECAM [22] and various glycolipids [23,24]. Platelets of adult blood group A_2_ individuals demonstrate minimal expression of A determinants [25], and adults of blood group A_1_ display a broad spectrum from low to high platelet A antigen expression. Strong expression of A and B antigens on platelets of some individuals was first described by Ogasawara et al. [13] and confirmed by others [12,26,27,28].The expression of A determinants is associated with the gene dose and genetic variants [14,29,30]. Representative fluorescence histograms of adult and neonatal platelets are shown in supplemental Figure S1.

In this study, we systematically investigated the expression of blood group A antigens on platelets of newborns. We demonstrated that the expression pattern mirrors that of adults, albeit on a lower level. In most newborns of blood group A, binding of monoclonal anti-A was detected on a subpopulation of platelets. Some newborns revealed high platelet expression of A determinants on all platelets (type II high-expressers). This phenotype was associated with neonatal thrombocytopenia in on case report [31]. We conclude that maternal anti-A (and/or anti-B) antibodies should also bind in vivo to a subset of antigen-positive fetal platelets in cases of fetal transplacental hemorrhage.

In HDFN, ABO incompatibility between the mother and fetus protects against primary D and non-D red blood cell alloimmunization by pregnancy (for review see [7]). D sensitization in RhD-negative women results from the passage of D-positive fetal red blood cells across the placenta into maternal circulation (for review see [32]). Chown was the first to demonstrate fetomaternal (macro) transfusion in an RhD-negative woman who had given birth to a baby with severe normoblastic anemia by detecting D-positive red blood cells in her circulation. She developed anti-D within three weeks of delivery [33]. Later, it was shown that transplacental (micro) hemorrhage is a regular phenomenon in pregnancy. In one study, the incidence of fetal transplacental hemorrhage was 3% in the first trimester, 12.1% in the second trimester, 45.5% in the third trimester, and 63.6% after delivery [34]. In this study, the amount of fetal transplacental hemorrhage ranged from 0.01 mL to 0.06 mL of fetal red blood cells already in the first and second trimesters, and the amount increased during the third trimester. In post-delivery samples, approximately 50% of women demonstrated ≥0.15 mL of circulating fetal red blood cells [34]. However, anti-D is rarely found in blood samples taken after delivery in RhD-negative primiparae giving birth to ABO-compatible RhD-positive babies [35]. The incidence of anti-D six months after delivery of an ABO-compatible RhD-positive baby is estimated to be 8.5%, and there is a direct relation between the amount of fetal red blood cells in the maternal circulation after delivery and the risk of immunization. A further 8.5% of mothers develop anti-D by the end of the second pregnancy, and it is postulated that these mothers had been primed by the first pregnancy (The incidence of anti-D six months after delivery of an ABO-incompatible RhD-positive baby in this study was 1%) [35]. Thus, the first pregnancy is usually not affected by HDFN, and immunization of pregnant women by fetal D-positive red blood cells occurs late in pregnancy and at delivery.

The protective effect of ABO incompatibility between the mother and child on anti-D immunization by pregnancy prompted Finn to suggest that it might be possible to destroy fetal red blood cells in the maternal circulation using a suitable antibody. This would prevent immunization mimicking the natural protection afforded by ABO incompatibility [5]. This suggestion led to the development of one of the most successful immunoprophylaxis therapies today, anti-D immunoglobulin: immunization of RhD-negative mothers was suppressed when gamma-globulin containing a high titer of incomplete anti-D was injected i.m. soon after delivery [36]. Today, anti-D immunoglobulin is the standard of care to prevent D immunization in pregnant women at risk.

The natural history of maternal immunization against D and HPA-1a exhibits two striking differences: (a) ABO incompatibility between the mother and fetus protects against immunization to red blood cell antigens (D and non-D antigens) but not against HPA-1a immunization by pregnancy (this study). (b) Primigravidae are immunized during pregnancy or at delivery against D without any consequences for the firstborn baby in most cases. Usually, clinically overt HDFN occurs only in a subsequent pregnancy (see above). In contrast, clinically overt FNAIT due to maternal anti-HPA-1a antibodies occurs regularly in primigravidae [37,38] and the fetuses of primigravidae can be severely affected by ICH. In a case series of 21 FNAIT cases with ICH, 71% (*n* = 15) occurred during the first-affected pregnancies as early as 18 weeks gestational age [38].

In the case of fetal transplacental hemorrhage, whole fetal blood, including platelets and leukocytes, is transferred to the maternal circulation [39]. Why does ABO incompatibility not protect against maternal anti-HPA-1a immunization? The expression of A and B blood group antigens on fetal platelets may be too low compared with the expression on red blood cells. However, A antigen expression on red blood cells of newborns is also weak compared with adult cells [40]. In one study, only 26% (median, *n* = 13) of newborn red blood cells of blood group A were agglutinated by Dolichos biflorus lectin compared with 94% (median) of adult A_1_ red blood cells [41]. Thus, the expression pattern of blood group A antigens on platelets and red blood cells of newborns is similar: the expression is weak compared with adult cells and absent or nearly absent on subpopulations.

To our knowledge, the mechanism of action of the protective effect of ABO incompatibility against anti-D immunization by pregnancy is unknown. Fetal red blood cells are also detectable at delivery in ABO-incompatible pregnancies, albeit at a lower incidence than in compatible pregnancies (in one series in 19% versus 50% of post-partum samples [42]). This result may be due to the absence of A and/or B antigens on a subpopulation of fetal red blood cells [41], which survive in the maternal circulation despite the presence of anti-A and/or anti-B antibodies. This finding makes it unlikely that all incompatible fetal red blood cells are destroyed by maternal IgM and/or IgG anti-A and/or anti-B antibodies, preventing their recognition by the immune system. In consequence, in ABO incompatibility, antibody-mediated immune suppression (AMIS; for review see [43]), mediated by maternal anti-A and/or anti-B IgG antibodies, may be causative of the protective effect. In animal models of AMIS, immunosuppressive IgG does not necessarily mask all epitopes, and red blood cell clearance does not mediate the immunosuppressive effect [43]. Furthermore, several animal models have shown that injection of presensitized (IgG coated) platelets could prevent alloimmunization (for review see [44]). If fetal platelets are the cellular source of HPA-1a antigens immunizing the mother via fetal transplacental hemorrhage,-like red blood cells in HDFN -, we would expect sensitization by maternal A and/or B antibodies and a protective effect of ABO incompatibility since at least a subpopulation of neonatal platelets expresses blood group A antigens. However, the research on the mechanism of action of AMIS in murine models came to different conclusions, and murine models of AMIS cannot be inevitably extrapolated to humans.

An alternative hypothesis is that the cellular source immunizing the pregnant mother against HPA-1a antigens are fetal cells that do not express A and/or B antigens, e.g., leukocytes or trophoblast cells. The entry of fetal trophoblast cells, lymphocytes, hematopoietic stem cells, and other fetal cells into the maternal circulation is a physiological phenomenon that occurs as early as 4–6 weeks in pregnancy (for review see [45]). Trophoblast cells express β3 integrin [46], carrying the HPA-1a/1b polymorphism, and lack A and B blood group antigen expression [47,48]. The early confrontation of primigravidae with an alloantigen on trophoblast cells or trophoblast cell debris may cause immunization against HPA-1a and FNAIT during the first pregnancy. The lack of A and B blood group antigens on trophoblast cells may explain the absence of protection against HPA-1a immunization by ABO incompatibility between the mother and fetus. The striking differences in the natural history of maternal immunization against D and HPA-1a should be considered in the development of anti-HPA-1a immunoprophylaxis in pregnant women at risk [49].

## Figures and Tables

**Table 1:** The ABO phenotype distribution between FNAIT cases (mothers or neonates) and controls did not differ (Chi-square test, *p* > 0.05).

ABO Phenotype	Cases (Mothers) (*n* = 118)	Cases (Neonates) (*n* = 118)	First Time Blood Donors (*n* = 45,295)
0	35%	39%	41%
A	47%	44%	42%
B	13%	10%	12%
AB	5%	7%	5%

## Data Availability

Requests for the deidentified data used in this study can be sent to the corresponding author, ending 24 months after publication of this article. The study protocol will be made available upon reasonable request to the corresponding author.

## Supplementary Materials

The following supporting information can be downloaded at: https://www.mdpi.com/article/10.3390/jcm11226811/s1, Table S1: Analysis of *ABO* genotyping TaqMan probes for redundancy. Figure S1: Fluorescence histograms of adult and neonatal platelets.
