# Supplementary material for: ABO Incompatibility between the Mother and Fetus Does Not Protect against Anti-Human Platelet Antigen-1a Immunization by Pregnancy
Source: J Clin Med. 2022 Nov 17;11(22):6811. doi: 10.3390/jcm11226811 (PMC9694632; doi:10.3390/jcm11226811)
Supplement: Supplementary file 1 [file jcm-11-06811-s001.zip › Supplemental_Figure_S1.pptx]

## Slide 1
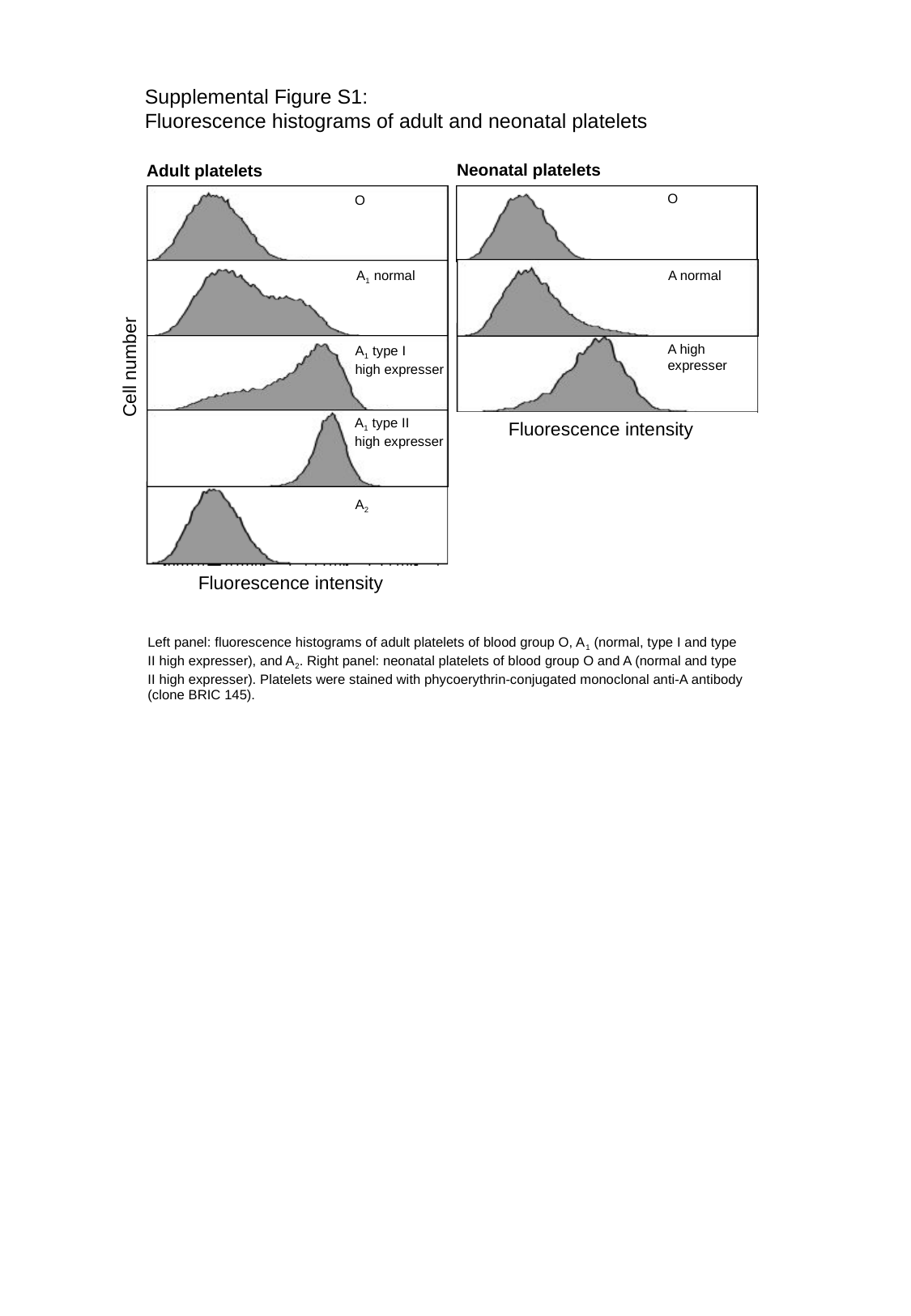

Supplemental Figure S1:
Fluorescence histograms of adult and neonatal platelets
Neonatal platelets
Adult platelets
O
O
A1 normal
A normal
A high
expresser
A1 type I
high expresser
A1 type II
high expresser
A2
Cell number
Fluorescence intensity
Fluorescence intensity
Left panel: fluorescence histograms of adult platelets of blood group O, A1 (normal, type I and type II high expresser), and A2. Right panel: neonatal platelets of blood group O and A (normal and type II high expresser). Platelets were stained with phycoerythrin-conjugated monoclonal anti-A antibody (clone BRIC 145).
