# Supplementary material for: ABO Incompatibility between the Mother and Fetus Does Not Protect against Anti-Human Platelet Antigen-1a Immunization by Pregnancy
Source: J Clin Med. 2022 Nov 17;11(22):6811. doi: 10.3390/jcm11226811 (PMC9694632; doi:10.3390/jcm11226811)
Supplement: Supplementary file 1 [file jcm-11-06811-s001.zip › Supplemental_Table_S1.pdf]

Supplemental Table S1.

Analysis of *ABO* genotyping TaqMan probes for redundancy

	ABO genotyping TaqMan probes							
Alleles	c.261G	c.261del	c.703G	c.703A	c.802G	c.802A	c.1061C	c.1061delC
*ABO*A1.01*	+	-	+	-	+	-	+	-
*ABO*A2.01*	+	-	+	-	+	-	-	+
*ABO*B.01*	+	-	-	+	+	-	+	-
*ABO*O.01*		+	+	-	+	-	+	-
*ABO*O.02*	+	-	+	-	-	+	+	-

We are indebted to Carlheinz Mueller, University of Ulm, Germany, who analyzed this set of probes for redundancy employing the MinProb algorithm: Analysis and Optimization of Sets of Oligonucleotide probes (32-bit-version, unpublished): all 5 alleles can be distinguished; all 15 genotypes can be distinguished; the set is irreducible.
